# Ultraprocessed Food Consumption and Pre-Farmgate Greenhouse Gas Emissions Among United States Adults from 2007–2010

**DOI:** 10.1016/j.cdnut.2025.107460

**Published:** 2025-05-07

**Authors:** Rhea Jayaswal, Sarah M Frank, Euridice Martinez Steele, Donald Rose, Lindsey Smith Taillie

**Affiliations:** 1 Carolina Population Center, University of North Carolina at Chapel Hill, Chapel Hill, NC, United States; 2 Center for Science in the Public Interest, Washington, DC, United States; 3 Department of Nutrition, Gillings School of Global Public Health, University of North Carolina at Chapel Hill, Chapel Hill, NC, United States; 4 Global Academy of Agriculture and Food Systems, Royal (Dick) School of Veterinary Medicine, The University of Edinburgh, Edinburgh, United Kingdom; 5 Center for Epidemiological Studies in Health and Nutrition, University of São Paulo, São Paulo, Brazil; 6 Department of Social, Behavioral, and Population Sciences, Celia Scott Weatherhead School of Public Health, Tulane University, New Orleans, LA, United States

**Keywords:** food processing, ultraprocessed foods, greenhouse gas emissions, dietary emissions, planetary health, environmental health, NHANES, dataFRIENDS

## Abstract

**Background:**

Changes to the food system can have consequences for human and planetary health, and one recent change is increased consumption of ultraprocessed foods (UPFs). Although correlations between UPF intake and human health have been evaluated in the United States, little is known about the association of UPF intake with pre-farmgate greenhouse gas emissions (GHGEs), which account for the majority of GHGE across the food life cycle.

**Objectives:**

We used a nationally representative survey to evaluate the association between UPF consumption and pre-farmgate GHGE among United States adults.

**Methods:**

Data were from the National Health and Nutrition Examination Survey, 2007–2010. Participants were divided into quintiles based on proportion of grams from UPF using mean of 2-d dietary recall. The database of Food Recall Impacts on the Environment for Nutrition and Dietary Studies was used to estimate the pre-farmgate GHGE (in kilograms of carbon dioxide equivalents [kg CO_2_-eq]) of foods. Multivariate linear regression models were used to test the association between quintiles of UPF consumption and pre-farmgate GHGE. Models were progressively adjusted for sociodemographic characteristics, total caloric intake, and red and processed meat intake.

**Results:**

Consuming a greater proportion of grams from UPF was associated with higher pre-farmgate GHGE in unadjusted analyses. However, when the analyses were adjusted for total energy, we saw the opposite trend, such that consuming a higher proportion of grams from UPF was associated with lower pre-farmgate emissions: emissions for the highest quintile of UPF consumption were 4.47 (95% confidence interval [95% CI]: 4.27, 4.67) kg CO_2_-eq, compared with 4.85 (95% CI: 4.64, 5.05) kg CO_2_-eq for the highest quintile (*P*_trend_ = 0.003).

**Conclusions:**

Although we find that diets with a greater proportion of grams from UPF have lower pre-farmgate GHGE, our analyses show an opposite trend when they are not adjusted for total energy intake, demonstrating the need for caution when analyzing the relationship of UPF intake with GHGE.

## Introduction

Current food systems are a key driver of both noncommunicable disease and climate change [[1], [2], [3], [4]]. The food system is responsible for over one-third of global greenhouse gas emissions (GHGEs), and most of these emissions come from the “pre-farmgate” or agricultural production stage of the food cycle, rather than stages, such as processing, packaging, and transportation [5]. When considering the joint health and environmental impacts of food, much of the literature has focused on animal-sourced foods because of the high environmental demand of livestock production and the high intake of red and processed meat in the United States [6,7]. To date, dietary recommendations to reduce the environmental burden of agricultural production, particularly in high-income nations, such as the United States, have largely centered on reducing meat, dairy, and other animal-sourced food consumption [[8], [9], [10], [11], [12], [13]].

However, there has been an increasing focus on ultraprocessed foods (UPFs) as a potential driver of health harms and a source of environmental damage. UPFs are typically defined as part of the Nova classification system, which categorizes foods by degree of processing into 4 mutually exclusive levels: *1*) unprocessed or minimally processed foods, *2*) processed culinary ingredients, *3*) processed foods, and *4*) UPFs. UPFs are food and drink formulations created from a series of industrial processes, made from ingredients of mostly industrial use, typically containing cosmetic additives (e.g., flavors, colors, emulsifiers), and often high in added sugar, sodium, and saturated fat [14]. Examples include soft drinks, packaged chips and cookies, and reconstituted meat products (or meat alternatives), such as nuggets and sausages.

The worldwide increase in UPF consumption is a major shift in the food system in recent decades [15]. In the United States, UPFs comprise over half of all kilocalories consumed [16], and UPF intake has steadily increased over the last 20 y [17,18]. Greater UPF intake has been linked with poor diet quality and many deleterious health outcomes, such as all-cause mortality, obesity, and several noncommunicable diseases, such as heart disease, type 2 diabetes, and certain cancers [[19], [20], [21], [22], [23]]. UPFs are accessible, affordable, and reflective of an industrialized and globalized food supply that is dominated by transnational corporations [24].

Although the health impacts of UPF have been well documented, the environmental and climate impacts of UPF have been relatively less explored [25,26]. One narrative review found that globally, UPF accounted for up to one-third of total dietary GHGE [27]. Studies from outside the United States—namely, in Brazil [28] and France [29]—have examined the association between UPF and dietary GHGE, but their methodologies differ in measuring exposure in kilocalories or grams and how they adjust for confounders (such as sociodemographic characteristics and total energy intake), which may explain inconsistencies in their results and highlights the complexity of how UPF may impact the environment. No studies to date have assessed the association of UPF consumption with GHGE in the United States. When considering the life cycle of foods from farm to fork, the majority of all GHGEs come from pre-farmgate stages, and thus it is important to understand pre-farmgate GHGE in the United States [5,28]. Understanding the link between UPF and pre-farmgate GHGE in the United States is important because the United States has the second highest level of GHGE globally, with 11% of all emissions coming from agriculture [29].

The objectives of this study are to test the association between UPF consumption reported in the National Health and Nutrition Examination Survey (NHANES) and pre-farmgate GHGE in the database of Food Recall Impacts on the Environment for Nutrition and Dietary Studies (dataFRIENDS) among United States adults. This study focuses on emissions from the agricultural production stage of the food cycle, and stages such as processing, packaging, and transport are excluded due to a lack of data. The rapid growth in UPF consumption necessitates further investigation into their environmental effects to better understand the role they should play in a transition to a healthy, sustainable food system.

## Methods

### Data source and study population

**FIGURE 1:**
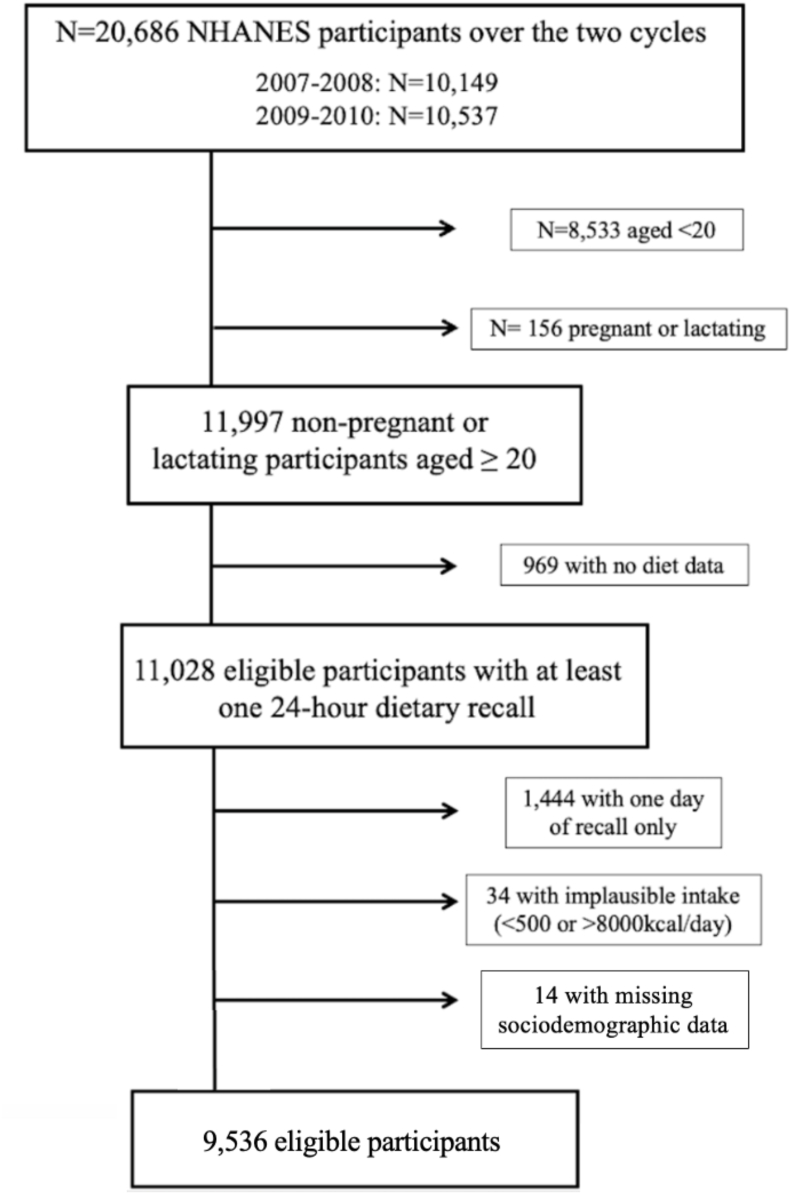
Flowchart of participant inclusion, National Health and Examination Survey, 2007–2008 and 2009–2010 cycles. NHANES, National Health and Nutrition Examination Survey.

Data were from the NHANES in years 2007–2008 and 2009–2010. These years were used because at the time of this study, the dataFRIENDS—a database linking NHANES food items to pre-farmgate GHGE—was available up to the 2009–2010 cycle of NHANES. DataFRIENDS enabled us to quantify emissions from foods in NHANES and construct our dependent variable, thus we needed it to align with the years in NHANES. NHANES is a repeated, cross-sectional survey that uses multistage probability design to sample the noninstitutionalized, civilian United States population [30]. It includes 2 24-h dietary recall interviews along with demographic, socioeconomic, and other health-related data. Trained dietary interviewers asked participants to recall all foods and beverages consumed the previous day using the validated Automated Multiple Pass Method [31]. This study uses the mean of 2 d of 24-h recall to better capture foods that are not eaten daily. The first dietary recall interview was in-person at a Mobile Examination Center, and the second interview was conducted by telephone 3–10 d later but never on the same day of the week as the first interview. Further details on study design, protocol, and data collection methods are described elsewhere [32]. The study population included individuals aged 20 y or older who were not pregnant or lactating and completed 2 valid dietary recalls (Figure 1). The final analytical sample included 9536 individuals.

Participants with extreme values for mean caloric intake (<500 kcal/d or >8000 kcal/d) were excluded [33].

### Food classification by degree of processing

Foods were classified according to Nova, which is based on degree of processing [14,34]. Nova splits foods into 4 mutually exclusive groups: *1*) unprocessed or minimally processed foods (such as fresh, dry, or frozen fruits, vegetables, grains, legumes, meat, fish, and milk), *2*) processed culinary ingredients (such as table sugars, oils, salt), *3*) processed foods (such as canned foods, simple breads, and cheese), and *4*) UPFs (such as sodas, prepackaged cookies, and chips) (Table 1) [34]. UPFs, the focus of this study, are defined as formulations of ingredients, mostly of exclusive industrial use, resulting from a series of industrial processes.

**TABLE 1:** The Nova classification system: definitions and examples^1^.

Nova level and definition	Examples
Level 1: Unprocessed or minimally processed foods. Foods that are directly obtained from plants or animals without any alteration, or foods that underwent cleaning, removal of inedible or unwanted parts, fractioning, grinding, drying, fermentation, pasteurization, cooling, freezing, or other processes that do not add substances to the original food.	• Natural, packaged, cut, chilled or frozen fruits and vegetables • Fresh, frozen, dried beef, pork, poultry, and other meat and fish • Rice • All types of beans
Level 2: Processed culinary ingredients. Substances extracted from unprocessed foods or from the nature itself using processes such as pressing, grinding, crushing, pulverizing, and refining.	• Plant oils • Coconut and animal fats (including butter and lard) • Table salt, table sugar, and honey
Level 3: Processed foods. Food products manufactured with the addition of salt or sugar or other substances of common culinary use, such as oil or vinegar, to unprocessed or minimally processed foods.	• Canned and bottled vegetables, legumes, or fruits • Salted, smoked, or cured meat or fish • Cheeses
Level 4: Ultraprocessed foods. Food products made up from several ingredients including sugar, oils, fats, and salt (generally in combination and in higher amounts than in processed foods) and food substances of no or rare culinary use.	• Soft drinks, energy drinks, sweetened juices, and powders for juices • Sausages, chicken and fish nuggets or sticks • Preprepared frozen dishes • Packaged snacks

1 For more details, please see table 1 in Steele et al. [34]. Obtained permission to include.

To assign foods into their respective groups, the food description and ingredient list were assessed for each NHANES food code. Food items were initially sorted into the 4 Nova groups and mutually exclusive subgroups within each Nova group based on the following 3 variables from the NHANES recall databases: Main Food Description, Additional Food Description, and Standard Reference (SR) Code Description. Nova classifications were then modified, if necessary, based on the following 2 variables: Combination Food Type and Source of Food. For all food items judged to be a handmade recipe (prepared from scratch), the classification was applied to the underlying SR codes. Further details and rationale on this process are described elsewhere [16,34].

### Exposures

We chose to model UPF consumption both as a percent of total grams and as a percent of total kilocalories to reflect that there are trade-offs to either method of conceptualizing UPF intake. Due to the association between kilocalorie intake (and nutrient intake more broadly) and health, dietary intake is usually modeled in kilocalories rather than in grams. However, foods are produced and sold in grams; moreover, some UPF products contain low or no kilocalories (e.g., diet soda, sugar-free candies). Thus, for the main analysis, UPF consumption was based on percentage of gram intake of UPF (Nova group 4) out of total gram intake. Participants were then divided into quintiles based on the proportion of dietary mass (in grams) from UPF (%grams). Quintile 1 contains individuals with the lowest proportion of dietary grams from UPF consumption (%grams), whereas quintile 5 contains individuals with the highest proportion.

Then, for sensitivity analyses, the percent of total kilocalories of food (%kcal) was used rather than percent of total grams, using the same procedure of dividing participants into quintiles based on proportion of dietary energy (in kilocalories) from UPF [28,35].

### Outcomes

GHGEs are a major contributor to global warming and serve as a climate change indicator [36]. The dataFRIENDS was used to assign pre-farmgate GHGE to as-consumed foods reported in the NHANES dietary recalls [[10], [11], [12],^37^]. GHGEs were measured as kilograms of carbon dioxide equivalents per person per day (kg CO_2_-eq/person/d) to put the global warming potential of various gases—such as carbon dioxide, methane, and nitrous oxide—on the same scale.

DataFRIENDS links individual food items reported in NHANES dietary recalls to pre-farmgate GHGE by first translating those foods into commodities with recipes provided in the Food Commodities Intake Database [38]. The 332 unique food commodities were then linked to their GHGEs using a review of food Life Cycle Assessment (LCA) literature. Altogether, this process linked pre-farmgate GHGE to over 6000 as-consumed foods reported in dietary recalls for adults in the 2005–2010 cycles of NHANES. Additional details about the creation of this database have been documented previously [12].

LCA studies examine the relevant stages of a supply chain for a specific food and account for the associated environmental impacts at each stage, for example, production, processing, or transportation. LCA studies that were used to create dataFRIENDS included only pre-farmgate emissions (i.e., production phase only). Therefore, the GHGE estimates only fully capture the agricultural production stage of UPF commodity ingredients and not the GHGE for their transformation from commodity ingredient to final product [5,12]. The creators of dataFRIENDS estimate that 27% of total food-related emissions are not captured because of the missing processing and packaging stages.

### Sociodemographic correlates

NHANES demographic variables are self-reported. Sociodemographic characteristics considered in this study are age, sex, education, income, and race/ethnicity. Age was categorized into 20–39 y, 40–59 y, and 60 y or older. Family poverty-to-income ratio (PIR) relates family income to its respective poverty threshold. Categories were based on SNAP eligibility: PIR ≤ 1.30, >1.30–3.50, and >3.50 [39]. Education was categorized as less than high school, high school degree or equivalent, some college, and college graduate or above. Race/ethnicity was categorized into 4 groups based on self-report: non-Hispanic White; non-Hispanic Black; Hispanic (which included people who identified as Mexican American or as other Hispanic); and Asian, Multiracial, and Other non-Hispanic race/ethnicities.

### Statistical analyses

In descriptive analyses, we reported the median and IQR of pre-farmgate GHGE because the distributions of mean grams consumed per person and pre-farmgate GHGE were skewed. We estimated the survey-weighted distribution of sociodemographic characteristics (age, sex, income, education, and race/ethnicity) by quintile of UPF consumption and used Pearson’s coefficient to test for the differences between sociodemographic groups. Furthermore, we estimated consumption of mutually exclusive food subgroups in the lowest and highest quintiles of UPF consumption (in %kcal, because food subgroups' data in %grams were unavailable).

We used multivariate linear regression models to test the association between proportion of dietary gram intake from UPF (in quintiles) and pre-farmgate GHGE (in kg CO_2_-eq/person/d). Model 1 was an unadjusted analysis. Model 2 was adjusted for sociodemographic characteristics (age, sex, education, income, and race ethnicity) to account for potential confounding. Model 3 was adjusted for sociodemographic characteristics and total caloric intake. Model 4 was adjusted for sociodemographic characteristics, total caloric intake, and red and processed meat intake in grams. We adjusted for total caloric intake and red and processed meat intake (in grams) to test whether these variables might be driving the association as a mediator. In sensitivity analyses, we re-ran all models using the proportion of dietary energy (kilocalories) from UPF as the exposure variable. All models were analyzed as a complete case analysis. To verify that the assumptions of linear regression were met, we assessed the distribution of the residuals for normality. As the distribution of the residuals was approximately normal, the assumptions for linear regression hold. We estimated the percent change by taking the difference between quintile 5 and quintile 1 and dividing by quintile 1.

All analyses conducted were in Stata SE 17.0 and used NHANES survey weights to account for complex survey design. The NHANES complex survey weights account for the complex survey design of NHANES, such as oversampling of some demographic groups, survey nonresponse, and poststratification adjustments to match total population counts from the Census Bureau, and a weighted sample may be interpreted as representative of the noninstitutionalized, civilian population of the United States [40]. All statistical tests were conducted at a significance level of *P* < 0.05.

## Results

The final sample included 9536 adults, and sociodemographic characteristics of the sample can be found in Table 2. The mean proportion of gram intake from UPF was 31.5% (95% confidence interval [95% CI]: 30.3, 32.6), and the median pre-farmgate GHGE was 3.9 kg CO_2_-eq/person/d (IQR: 2.5 kg CO_2_-eq, 5.9 kg CO_2_-eq).

**TABLE 2:** Characteristics of Sample for United States adults aged ≥ 20 y, National Health and Nutrition Examination Survey 2007–2010 (*n* = 9536)^1^.

	Total
Age (y) % (*n*)	
20–39	30.8 (2943)
40–59	33.5 (3195)
≥60	35.7 (3412)
Sex % (*n*)	
Male	48.4 (4627)
Female	51.6 (4923)
Income^2^ % (*n*)	
PIR≤1.30	28.5 (2720)
PIR>1.30-3.50	34.9 (3337)
PIR>3.50	27.9 (2666)
Missing	8.7 (827)
Education % (*n*)	
Less than high school	28.1 (2675)
High school degree or equivalent	23.9 (2277)
Some college	27.5 (2621)
College graduate or above	20.6 (1963)
Race/ethnicity % (*n*)	
Non-Hispanic White	49.7 (4742)
Non-Hispanic Black	19.0 (1817)
Hispanic	27.4 (2615)
Asian, Multiracial, and Other Non-Hispanic	3.9 (376)
Mean share of diet from ultraprocessed food (% of grams /person/d) (95% confidence interval)	31.5 (30.3, 32.6)
Median dietary greenhouse gas emissions (kilograms CO_2_-eq/person/d) (IQR)	3.9 (2.5, 5.9)

1 Values are survey-weighted proportion (unweighted *N*) unless otherwise noted.

2 PIR refers to family poverty–income ratio.

UPF consumption varied widely across quintiles and by several sociodemographic characteristics. People who were 20–30 y old, male, had a PIR ≤ 1.30, had less than high school education, had a high school degree or equivalent, had some college, who were non-Hispanic White, and who were non-Hispanic Black were more likely to be in the highest quintile of UPF intake than in the lowest quintile (Table 3). However, people who were 60 y or older, female, college graduates, who were Hispanic, and who identified as other non-Hispanic race/ethnicity were more likely to be in the lowest quintile of UPF intake. We did not observe any other sociodemographic correlations with UPF intake.

**TABLE 3:** Distribution of sociodemographic characteristics by quintile of dietary share of ultraprocessed foods, National Health and Nutrition Examination Survey 2007–2010 (*n* = 9536)^1^.

	Quintile of dietary share of ultraprocessed foods (% of total gram intake)					*χ*^2^*P*
	Q1	Q2	Q3	Q4	Q5	
Age (y)						
20–39	15.1 (12.7, 17.8)	15.9 (14.1, 17.9)	18.0 (15.6, 20.7)	23.1 (20.7, 25.7)	28.0 (25.0, 31.1)	<0.0001
40–59	19.1 (16.8, 21.7)	19.3 (17.2, 21.5)	19.6 (17.7, 21.6)	20.5 (18.6, 22.6)	21.6 (18.7, 24.8)	
≥60 y	24.6 (22.3, 27.1)	25.1 (22.9, 27.4)	21.8 (19.6, 24.3)	17.1 (14.9, 19.5)	11.4 (9.7, 13.4)	
Sex						
Male	17.3 (15.4, 19.4)	17.6 (15.6, 19.9)	21.4 (19.9, 23.1)	21.9 (20.0, 23.9)	21.8 (19.6, 24.2)	0.0003
Female	20.6 (18.4, 23.0)	21.2 (19.4, 23.0)	17.8 (16.2, 19.7)	19.4 (17.9, 21.1)	21.0 (18.9, 23.4)	
Income^2^						
PIR≤1.30	18.2 (16.0, 20.6)	15.7 (13.9, 17.6)	16.3 (14.4, 18.4)	20.9 (18.3, 23.6)	29.0 (25.5, 32.7)	0.0001
PIR>1.30-3.50	18.4 (16.4, 20.7)	19.9 (18.3, 21.7)	19.5 (17.1, 22.2)	21.3 (19.2, 23.6)	20.8 (18.0, 23.9)	
PIR>3.50	19.1 (16.5, 22.0)	21.4 (18.7, 24.3)	20.5 (18.4, 22.8)	20.0 (17.9, 22.4)	19.0 (16.3, 22.2)	
Missing	23.5 (18.5, 29.2)	17.0 (13.3, 21.4)	23.5 (19.5, 28.1)	19.5 (15.9, 23.8)	16.5 (13.1, 20.7)	
Education						
Less than high school	17.4 (15.6, 19.3)	17.3 (15.4, 19.3)	20.3 (18.4, 22.3)	21.4 (19.1, 24.0)	23.6 (20.5, 27.1)	<0.0001
High school degree or equivalent	15.9 (13.6, 18.5)	16.2 (14.4, 18.2)	19.8 (16.7, 23.2)	21.5 (18.9, 24.4)	26.6 (23.3, 30.3)	
Some college	18.7 (17.2, 20.3)	19.6 (17.6, 21.7)	19.0 (16.9, 21.2)	21.0 (18.5, 23.7)	21.7 (19.4, 24.2)	
College graduate or above	23.1 (20.1, 26.5)	23.7 (20.4, 27.4)	19.5 (17.1, 22.1)	18.8 (16.2, 21.7)	14.9 (12.8, 17.3)	
Race/ethnicity						
Non-Hispanic White	18.6 (16.6, 20.8)	20.3 (18.4, 22.2)	19.6 (17.9, 21.4)	20.1 (18.7, 21.6)	21.4 (18.9, 24.2)	<0.0001
Non-Hispanic Black	14.1 (12.0, 16.6)	15.7 (13.4, 18.2)	17.5 (15.6, 19.7)	25.0 (21.7, 28.5)	27.7 (24.8, 30.9)	
Hispanic	19.3 (16.8, 22.1)	18.5 (16.6, 20.5)	23.1 (21.0, 25.3)	21.6 (18.7, 24.7)	17.6 (15.0, 20.6)	
Asian, Multiracial, and Other Non-Hispanic	33.1 (28.2, 38.3)	19.8 (13.9, 27.5)	14.6 (10.6, 19.8)	15.7 (10.8, 22.2)	16.9 (12.3, 22.7)	

1 Values are survey-weighted proportion (95% confidence interval) unless otherwise noted.

2 PIR refers to family poverty–income ratio.

The mean share of dietary grams from UPF was 61.7% (60.7%, 62.8%) of total grams consumed in quintile 5, compared with 8.8% (95% CI: 8.5, 9.1) in quintile 1 (Table 4). Consumption of Nova food subgroups differed between the highest and lowest quintile of UPF (%kcal) consumption (Table 5). For individuals in the highest UPF consumption group (quintile 5), the top contributors to total energy intake were ultraprocessed bread (12.2%), carbonated soft drinks (7.8%), and pizza (6.3%). In contrast, for individuals in the lowest UPF consumption quintile (quintile 1), the top contributors to total energy intake were minimally processed meat/poultry (12.9%), the “other” processed foods category (8.9%), which includes salted or sugared nuts and seeds, peanut or almond butter or spread, beer, and wine, and minimally processed fruits/juices (7.8%).

**TABLE 4:** Mean consumption of Nova levels (%grams) by quintile of dietary share of ultraprocessed foods, National Health and Nutrition Examination Survey 2007–2010 (*n* = 9536)^1^.

	Quintile of dietary share of ultraprocessed foods (% of total gram intake) (95% CI)				
	Q1	Q2	Q3	Q4	Q5
Nova level of processing					
Level 1: unprocessed or minimally processed foods	82.6 (81.8, 83.4)	74.8 (73.8, 75.8)	65.8 (65.2, 66.4)	55.7 (55.2, 56.2)	34.1 (33.1, 35.1)
Level 2: processed culinary ingredients	0.7 (0.6, 0.7)	0.7 (0.7, 0.8)	0.7 (0.6, 0.7)	0.7 (0.6, 0.7)	0.6 (0.5, 0.6)
Level 3: processed foods	7.9 (7.1, 8.8)	6.8 (5.8, 7.7)	6.7 (6.0, 7.4)	5.3 (4.8, 5.8)	3.6 (3.3, 3.9)
Level 4: ultraprocessed foods	8.8 (8.5, 9.1)	17.7 (17.5, 17.9)	26.8 (26.5, 27.0)	38.4 (38.1, 38.6)	61.7 (60.7, 62.8)

1 Values are survey-weighted proportion (95% confidence interval).

**TABLE 5:** Mean consumption of Nova food subgroups (%kcal) according to quintile of dietary share of ultraprocessed foods, National Health and Nutrition Examination Survey 2007–2010 (*n* = 9536)^1^.

	Quintile of dietary share of ultraprocessed foods (% of total energy intake) (95% CI)	
	Q1	Q5
Food subgroup Nova level 1		
Legumes	1.9 (1.6, 2.2)	0.3 (0.2, 0.3)
Roots and tubers	2.9 (2.6, 3.2)	0.9 (0.8, 1.0)
Vegetables	1.5 (1.4, 1.6)	0.5 (0.5, 0.5)
Fruit/juices	7.8 (7.2, 8.4)	2.3 (2.0, 2.6)
Meat/poultry	12.9 (12.1, 13.7)	5.5 (5.1, 5.9)
Fish	1.6 (1.3, 1.9)	0.4 (0.3, 0.5)
Eggs	2.2 (2.0, 2.4)	0.9 (0.8, 1.0)
Milk and yogurt	5.1(4.6, 5.5)	2.5 (2.3, 2.8)
Grains	7.3 (6.2, 8.4)	0.8 (0.7, 1.0)
Pasta	2.3 (2.0, 2.7)	1.1 (0.8, 1.3)
Other Nova level 1	4.1 (3.8, 4.5)	1.0 (0.9, 1.1)
Nova level 2		
Table sugar	1.8 (1.6, 2.1)	0.9 (0.7, 1.0)
Oils	2.3 (2.1, 2.5)	0.4 (0.3, 0.4)
Animal fats	1.5 (1.3, 1.7)	0.6 (0.6, 0.7)
Other Nova level 2	0.2 (0.1, 0.2)	<0.1 (<0.1, <0.1)
Nova level 3		
Cheese	3.2 (2.9, 3.6)	2.0 (1.8, 2.2)
Ham and salted meats	1.3 (1.1, 1.5)	0.8 (0.7, 0.9)
Plant foods in brine	1.1 (0.9, 1.2)	0.5 (0.4, 0.5)
Other Nova level 3	8.9 (8.1, 9.7)	1.6 (1.5, 1.8)
Nova level 4		
Reconstituted meat or fish products	1.2 (1.0, 1.4)	4.9 (4.5, 5.2)
Bread	7.5 (6.9, 8.0)	12.2 (11.6, 12.8)
Cookies, cakes, pies, pancakes, and pastries	2.0 (1.8, 2.3)	5.8 (5.2, 6.3)
Ice cream, ice pops, and frozen yogurts	1.1 (0.9, 1.4)	2.7 (2.3, 3.2)
Desserts and other sugary products	0.6 (0.5, 0.8)	1.0 (0.9, 1.2)
Sugared breakfast cereals	1.9 (1.6, 2.2)	2.8 (2.4, 3.3)
Salty snacks (chips, crackers, popcorn)	2.1 (1.8, 2.3)	6.0 (5.5, 6.5)
Sweet snacks	1.1 (1.0, 1.3)	3.2 (2.7, 3.7)
Frozen and shelf-stable plate meals	0.9 (0.6, 1.1)	4.2 (3.6, 4.7)
Pizza	0.5 (0.2, 0.7)	6.3 (5.5, 7.1)
Sandwiches and hamburgers on bun	0.1 (0.1, 0.2)	2.7 (2.3, 3.1)
French fries and potato products	0.4 (0.3, 0.5)	2.8 (2.5, 3.1)
Instant and canned soups	0.7 (0.6, 0.8)	0.8 (0.6, 1.0)
Sauces, dressings, gravy	2.4 (2.1, 2.6)	3.4 (3.2, 3.7)
Sugared milk drinks	1.0 (0.7, 1.3)	1.9 (1.4, 2.3)
Soft drinks, carbonated	1.3 (1.0, 1.5)	7.8 (6.7, 8.9)
Other sweetened beverages	1.3 (1.1, 1.6)	4.1 (3.7, 4.6)
Other Nova level 4	3.9 (3.6, 4.3)	4.3 (4.0, 4.7)

1 Values are mean survey-weighted proportion (95% confidence interval).

In the unadjusted linear regression, Model 1, there was a significant positive linear association between UPF (in %grams) and pre-farmgate GHGE (*P*_trend_ < 0.001, Table 6). In Model 1, the estimated emissions for quintile 5 were 4.76 (95% CI: 4.53, 5.00) kg CO_2_-eq, 11% higher than emissions for quintile 1 (4.29 [95% CI: 4.02, 4.56] kg CO_2_-eq; Table 6). The direction and statistical significance of this relationship was consistent in Model 2, which was adjusted for sociodemographic characteristics. However, in Model 3, upon adjustment for total energy intake, the relationship reversed, such that higher UPF intake (in %grams) was associated with lower pre-farmgate GHGE (*P*_trend_ = 0.003, Table 6). In Model 3, the estimated emissions after adjusting for total caloric intake and sociodemographic characteristics for quintile 5 were 4.47 (95% CI: 4.27, 4.67) kg CO_2_-eq, 8% lower than the estimates for those in quintile 1 (4.85 [95% CI: 4.64, 5.05] kg CO_2_-eq [*P*_trend_ = 0.003, Table 6]). Further adjusting for red and processed meat intake (*P*_trend_ < 0.001) in Model 4 yielded robust results to Model 3 (Table 6).

**TABLE 6:** Mean greenhouse gas emissions according to quintiles of the dietary contribution of ultraprocessed foods, National Health and Nutrition Examination Survey 2007–2010 (*n* = 9536)^1^.

	Quintile 1	Quintile 2	Quintile 3	Quintile 4	Quintile 5	*P*_trend_
Variable specification: exposure = proportion of total gram intake from ultraprocessed food						
Model 1: Unadjusted	4.29 (4.02, 4.56)	4.44 (4.27, 4.62)	4.68 (4.49, 4.88)	4.78 (4.56, 5.00)	4.76 (4.53, 5.00)	0.001
Model 2: Model 1 + sociodemographic characteristics^2^	4.42 (4.17, 4.66)	4.56 (4.38, 4.74)	4.60 (4.40, 4.80)	4.70 (4.46, 4.94)	4.70 (4.47, 4.92)	0.033
Model 3: Model 2 + total caloric intake	4.85 (4.64, 5.05)	4.60 (4.43, 4.76)	4.61 (4.46, 4.76)	4.50 (4.26, 4.73)	4.47 (4.27, 4.67)	0.003
Model 4: Model 3 + red and processed meat intake^3^	4.99 (4.88, 5.10)	4.61 (4.49, 4.73)	4.58 (4.45, 4.70)	4.53 (4.39, 4.67)	4.33 (4.19, 4.47)	<0.001
Variable specification: exposure = proportion of total caloric intake from ultraprocessed food						
Model 1: unadjusted	4.79 (4.48, 5.09)	4.83 (4.61, 5.04)	4.84 (4.63, 5.04)	4.48 (4.28, 4.68)	4.14 (3.98, 4.29)	<0.001
Model 2: Model 1 + sociodemographic characteristics^2^	4.78 (4.49, 5.07)	4.86 (4.64, 5.08)	4.83 (4.64, 5.03)	4.51 (4.30, 4.72)	4.09 (3.93, 4.26)	<0.001
Model 3: Model 2 + total caloric intake	5.06 (4.83, 5.29)	4.90 (4.70, 5.10)	4.77 (4.63, 4.92)	4.44 (4.27, 4.62)	3.93 (3.81, 4.06)	<0.001
Model 4: Model 3 + red and processed meat intake^3^	5.06 (4.92, 5.20)	4.87 (4.75, 4.99)	4.69 (4.61, 4.78)	4.36 (4.24, 4.47)	4.11 (4.01, 4.21)	<0.001

1 Values are survey-weighted proportion (95% confidence interval) unless otherwise noted.

2 Sociodemographic characteristics include age, education, income, sex, and race/ethnicity.

3 Red and processed meat intake was measured in grams.

In sensitivity analyses, we redefined UPF consumption as proportion of total daily kilocalories of food intake. In Model 1, the unadjusted analyses, there was a significant inverse association between UPF (in %kcal) and pre-farmgate GHGE (*P*_trend_ < 0.001, Table 6). In Model 1, estimated emissions for quintile 5 were 4.14 (95% CI: 3.98, 4.29) kg CO_2_-eq, 14% lower than estimated emissions for quintile 1 (4.79 [95% CI: 4.48, 5.09] kg CO_2_-eq [*P*_trend_ < 0.001, Table 6]). This trend was robust after adjusting for sociodemographic characteristics, total caloric intake, and red and processed meat intake (*P*_trend_ < 0.001, Table 6) with the inverse relationship between UPF and GHGE strengthening in the models adjusted for total caloric intake.

## Discussion

The main findings of our study were that higher gram intake from UPF is associated with lower pre-farmgate GHGE, although this relationship is dependent on how UPF exposure is defined (in grams compared with kilocalories) and whether analyses were adjusted for total energy intake, which may be a potential mediator of the association.

In this nationally representative sample of United States adults, individuals in the highest quintile of UPF intake (in %grams) had 8% lower estimated pre-farmgate GHGE than those in the lowest quintile. Contrary to conceptual models posed by other articles, this suggests that diets high in UPF are less carbon intensive in the production stage than diets with less UPF. There are several possible explanations for this result. First, UPFs are high in refined grains, which contribute relatively fewer GHGE than animal-sourced foods, such as beef, pork, dairy, or cheese, none of which are considered UPF but all of which have relatively high GHGE. Indeed, ultraprocessed bread was the greatest contributor to total caloric intake among individuals in the highest quintile of UPF intake, and in contrast, minimally processed meat and poultry were the biggest contributors to total caloric intake among individuals in the lowest quintile of UPF intake. Although these differences are small, this could partially explain why lower UPF intake was associated with greater pre-farmgate GHGE. However, lower UPF intake was still associated with higher pre-farmgate GHGE even after adjusting for red and processed meat intake, indicating that differences in red and processed meat intake do not fully explain the inverse relationship between quintile of UPF intake and pre-farmgate GHGE. Second, our data only include “seed-to-farm gate” GHGE. We cannot account for other important aspects unique to UPF that may affect their GHGE, including the production of additives, such as artificial colorings, flavorings, or stabilizers, as well as the processing, packaging, and transportation steps of the food cycle. All these steps would likely increase the overall carbon footprint of UPF and are theorized to have other negative environmental externalities [26,27].

We found that the specification of UPF intake—i.e., whether it is defined as proportion of energy or proportion of grams—and adjustment for total energy intake can lead to different conclusions on the estimated environmental impacts of these foods. In unadjusted analyses of UPF as a percent of grams consumed, higher UPF intake was associated with greater pre-farmgate GHGE, but the association is reverse when defining UPF as a percent of kilocalories consumed or when adjusting for total caloric intake. This variation may be attributed to the diverse characteristics of UPF, such that there are UPFs that are light in weight but high in kilocalories, such as corn chips, and there are also UPFs that are heavy and low in kilocalories, such as diet soft drinks. To our knowledge, only 2 other nationally representative studies previously examined the relationship between UPF intake and GHGE, but these studies define exposure and adjust for confounders differently and thus are difficult to compare [28,41]. Both studies found that higher UPF intake was associated with greater GHGE but only in unadjusted analyses. In France, the exposure was defined as %grams from UPF, and the positive relationship was erased after adjusting for total energy intake [28]. In Brazil, the exposure was defined as %kcal from UPF, and the positive relationship was erased after adjusting for sociodemographic characteristics [41]. The varied definitions, conceptualizations, and findings across study and country context suggest that consistent definitions of UPF and consistent measurement of their production-related emissions are needed to best understand the association between UPF and GHGE across the globe.

Our analyses with UPF intake defined in proportion of grams indicate that total caloric intake is a mediator in the positive relationship between UPF intake and pre-farmgate GHGE. The positive relationship between UPF intake and pre-farmgate GHGE was reversed after adjusting for total caloric intake. This reversal suggests that higher total caloric intake correlated to higher share of diet from UPF, driving the positive association between UPF intake (in %grams) and pre-farmgate GHGE. Our unadjusted results are similar to the study in France, which found a positive association between UPF consumption (in %grams) and GHGE, but as in our study, this did not remain significant after adjusting for total caloric intake, again suggesting that greater dietary share of UPF (in %grams) increases GHGE by increasing total energy intake [28]. Additionally, recent studies from the Netherlands [42] and Brazil [41] found that UPF consumption contributed relatively less GHGE than minimally processed food intake, implying that on a calorie basis, they have a lesser impact than non-UPF foods.

Regardless of our findings, research shows that UPFs are a growing contributor to dietary GHGE. This is at least partially because UPF consumption has increased—over half of kilocalories on mean in our sample come from UPF. And in tandem with the increasing prominence of UPF in diet, UPFs have also contributed more to dietary emissions over time: a study in Brazil found that GHGE from UPF increased by 245% over the last 3 decades [43]. Researchers have theorized that greater UPF intake may increase GHGE because of reliance on high-emission insecticides, pesticides, and fertilizers to maintain large-scale monoculture farms, as well as postfarm stages involving more processing steps, more packaging, and longer transport distances [44].

It is crucial to note that most UPFs—and therefore, UPF-associated emissions—are not biologically “essential” for maintaining life; high UPF intake is in fact correlated with negative health outcomes. Therefore, public health efforts to reduce UPF consumption could represent a “win–win” for health and environmental outcomes [21,22]. In addition to accounting for a high proportion of dietary GHGE, UPF intake has been correlated with increased risk of obesity, type 2 diabetes, certain cancers, and cardiovascular disease [[19], [20], [21], [22]]. Reducing the share of UPF in the diet could simultaneously address UPF-related GHGE and lessen the risk of these health outcomes. Additionally, reducing UPF would reduce the production and environmental costs associated with industrial additives and packaging, as well as reduce public exposure to these substances, some of which are potential carcinogens [26]. It is important to note that the type of substitution for UPF matters; substituting UPF for their less-processed analog (e.g., natural yogurts or breads with no additives) would have health and environmental cobenefits, whereas substituting UPF for less-processed emission-intensive foods such as red meat could increase the overall carbon footprint of diets. Another key consideration is the relative affordability and accessibility of UPF, which provides inexpensive kilocalories and convenience for millions of busy families. We observed higher UPF intake among adults who were younger, male, had lower incomes, had lower educational attainment, and who were identified as non-Hispanic Black, consistent with previous findings in the United States [45]. A transition to diets lower in UPF and higher in minimally processed foods must be accompanied by policies that account for the true health and environmental cost of UPF and increase the accessibility and affordability of their less-processed counterparts.

The present study has several strengths and limitations. The use of NHANES allowed for nationally representative estimates of UPF intake and pre-farmgate GHGE. However, the dietary survey data were collected from 24-h recall, which is subject to measurement error and recall bias, and which does not represent usual intake. Of note, although there is measurement error for individuals, 24-h recall data provide valid estimates of population-level intake. Additionally, NHANES collects some information indicative of food processing such as place of meals and product brands, but the data are not consistently determined for all food items, which could lead to inaccuracies in UPF estimates. NHANES also does not specify production methods or geographical origin of consumed food, so estimated pre-farmgate GHGE cannot account for factors such as the difference in either the emissions between organic and conventionally produced foods or the emissions associated with food transport.

Indeed, as noted earlier, dataFRIENDS accounted for emission impacts from agricultural production but did not include impacts from processing beyond the farmgate [12]. Although most diet-related GHGEs are from the agricultural production stage [5,12,28], this limitation may cause an underestimate of emissions from foods that have additional processing, packaging, and transportation steps. Post-farmgate emissions are estimated to contribute toward 27%–43% of total emissions from the food system [5,12]. On one hand, this could be of particular concern for UPFs, which by definition undergo extensive processing and often have single-use packaging. Although including additional post-farmgate steps would increase estimated emissions from UPF, there may be similar packaging, processing, and transportation steps for most non-UPF as well. As an example, researchers in France found that post-farmgate emissions account for 27% of total emissions for people who consume diets low in UPF, and post-farmgate emissions account for 25% of total emissions for people who consume diets high in UPF—similar in both groups. Therefore, we do not anticipate that adding post-farmgate emissions would substantively change the conclusions of the present study, because the majority of emissions in agriculture stem from the production phase of food [5], but updated LCA that account for post-farmgate emissions, incorporating all stages from seed to postconsumer waste, are needed to confirm this hypothesis and to better understand how emissions for UPF and non-UPF may differ across their life cycles.

Finally, it is important to note that although our study only considers GHGE, there are other important environmental outcomes relevant to food production, such as energy use, water use, land use, and biodiversity loss, which high UPF diets may exacerbate [27]. Studies in France and Brazil have assessed correlations between greater UPF intake and some of these environmental indicators; in particular, the study in Brazil found a positive association between UPF intake (in %kcal) and water use, and the study in France found a positive association between UPF intake (in %grams) and land use, although both these were attenuated after adjusting for energy intake [28,41].

In conclusion, ours is the first study to show that among United States adults, diets with a high proportion of energy from UPF tend to have lower pre-farmgate GHGE. We add to a global body of work seeking to understand the relationship between UPF intake and dietary GHGE and find substantial heterogeneity in the results based on whether UPFs are measured as a proportion of dietary energy or dietary grams and whether adjusting for total energy intake. Further research is needed to understand the underlying mechanisms of these associations.

## Author contributions

The authors’ responsibilities were as follows — SMF, RJ, LST: designed research; DR, EMS, LST: provided materials. RJ, SMF: conducted research and analyzed data; RJ, SMF, EMS, DR, LST: wrote the paper; LST: had primary responsibility for final content; and all authors read and approved the final manuscript.

## Data availability

The NHANES data described in the manuscript, code book, and analytic code are publicly and freely available without restriction at https://wwwn.cdc.gov/nchs/nhanes/default.aspx. The dataFRIENDS data described in the manuscript, code book, and analytic code will be made available upon request pending application and approval. A related data set and codebook, the database of Food Impacts on the Environment for Linking to Diets (dataFIELD) is publicly and freely available without restriction from the University of Michigan Center for Sustainable Food Systems at https://css.umich.edu/research/data-downloads.

## Funding

Wellcome Trust. Grant Number: 216042/Z/19/Z to Lindsey Smith Taillie. The funding source had no role in the study design; the collection, analysis and interpretation of data; the writing of the report; or the decision to submit for publication.

## Conflict of interest

The authors report no conflicts of interest.
